# Comparison of piezosurgery vs. conventional bone chisel for extraction of impacted mandibular third molars with root–canal intimacy: a retrospective cohort study with CBCT-based preoperative planning

**DOI:** 10.3389/fmed.2026.1950396

**Published:** 2026-10-16

**Authors:** Huijun Hu, Yuchao Zhang, Jian Tan, Yang Yu

**Affiliations:** 1 The Second Department, The Affiliated Stomatological Hospital of Nanjing Medical University, Nanjing, Jiangsu, China; 2 State Key Laboratory Cultivation Base of Research, Prevention and Treatment for Oral Diseases, Nanjing Medical University, Nanjing, Jiangsu, China; 3 Jiangsu Province Engineering Research Center of Stomatological Translational Medicine, Nanjing Medical University, Nanjing, Jiangsu, China; 4 Department of Oral and Maxillofacial Surgery, The Affiliated Stomatological Hospital of Nanjing Medical University, Nanjing, Jiangsu, China; 5 Department of Stomatology, The First Affiliated Hospital of Changsha Medical University, Changsha, Hunan, China; 6 The Third Department, The Affiliated Stomatological Hospital of Nanjing Medical University, Nanjing, Jiangsu, China

**Keywords:** cone-beam computed tomography, impacted mandibular third molar, inferior alveolar nerve canal, minimally invasive tooth extraction, ultrasonic bone knife

## Abstract

**Objective:**

To compare clinical outcomes of piezosurgery vs. conventional chisel for extracting impacted mandibular third molars (IMTMs) with roots adjacent to the inferior alveolar nerve canal (IANC) under identical CBCT-based planning, isolating the ultrasonic osteotome's incremental benefit beyond shared imaging.

**Methods:**

A retrospective comparative cohort study was conducted using the clinical data of 126 patients with IMTMs whose tooth roots were closely adjacent to the IANC and who were treated in the Department of Oral and Maxillofacial Surgery at our hospital between September 2025 and May 2026. All patients underwent CBCT three-dimensional reconstruction before the operation to evaluate the anatomical structure and formulate the surgical plan. The patients were divided into the piezosurgery (*n* = 63) and conventional surgery (*n* = 63) groups. The conventional surgery group underwent traditional bone chiseling and crown splitting for extraction, while the piezosurgery group underwent extraction using the ultrasonic bone knife minimally invasive technique. The operation time, intraoperative blood loss, pain visual analogue scale (VAS) scores at different time points after surgery, degree of facial swelling, degree of mouth opening limitation, and complication occurrence rate were compared between the two groups.

**Results:**

Compared with the conventional surgery group, the piezosurgery group exhibited a markedly shorter surgical procedure duration and a significantly reduced volume of intraoperative blood loss, with all between-group comparisons yielding statistically significant results (*P* < 0.05). There were interaction effects between the two groups and at different time points in the VAS scores, facial swelling differences, and mouth opening limitation differences (*P* < 0.05). The VAS scores, facial swelling differences, and mouth opening limitation differences in the piezosurgery group at 1 day, 3 days, and 7 days after surgery were all lower than those in the conventional surgery group (*P* < 0.05). Additionally, the incidence of adverse postoperative complications was markedly lower in the piezosurgery group than in the conventional surgery group, and this discrepancy was statistically significant (*P* < 0.05).

**Conclusion:**

The CBCT-guided ultrasonic osteotome minimally invasive technique shortens operative time, reduces bleeding, alleviates postoperative pain, swelling, and trismus, and lowers complication rates in IMTM extraction with root-canal proximity.

## 1 Introduction

Impacted mandibular third molars (IMTM) rank among the most prevalent clinical disorders encountered in oral and maxillofacial surgery, and surgical removal of these teeth is among the most routinely performed operative procedures in daily dental clinical practice (1). When the root of the IMTM is in close contact with or partially encircles the inferior alveolar nerve canal (IANC), the traditional bone chiseling and crown splitting method, owing to unclear vision and blind operation, is prone to causing injury to the inferior alveolar nerve, resulting in serious complications such as numbness and abnormal sensation of the lower lip after surgery (2, 3). Furthermore, conventional surgical approaches tend to inflict substantial surgical trauma on patients, typically triggering severe postoperative discomfort, including intense pain, facial edema, and trismus. These adverse postoperative symptoms can greatly compromise patients' daily functioning and overall quality of life after treatment (4). As an advanced imaging modality applied in clinical diagnosis, cone-beam computed tomography (CBCT) delivers fine spatial resolution and relatively rapid scanning. However, its effective radiation dose (approximately 26–107 µSv) is higher than that of panoramic radiography (approximately 4.7–14.9 µSv) but substantially lower than that of medical CT (approximately 500–1,000 µSv). Therefore, its use is justified only in cases where additional anatomical information is clinically necessary, such as the high-risk root-canal proximity scenario in the present study. Its three-dimensional reconstruction technology can clearly and stereoscopically display the position, shape, and number of tooth roots, and the three-dimensional spatial relationship between the IMTM and IANC, providing a reliable basis for preoperative precise assessment and surgical plan formulation (5, 6). However, based on the same preoperative assessment, different surgical methods still have significant differences in the degree of intraoperative trauma, postoperative reactions, and risk of nerve injury. The piezoelectric osteotome is a novel class of surgical instruments designed specifically for bone-cutting procedures. It uses high-intensity focused ultrasound energy to selectively remove bone tissue without damaging the soft tissues and nerve vessels. It has precise cutting, less heat generation, and a clear surgical field compared to other methods. This surgical instrument has gained extensive clinical application in oral and maxillofacial surgery (7). However, most current studies separately examine these two technologies or do not strictly separate the synergistic effect of imaging methods and surgical instruments, with insufficient variable control, making it difficult to accurately evaluate the net benefit of the combined technology (8, 9). Accordingly, this study strictly adhered to the principle of controlling for single variables. All included patients underwent a unified standard CBCT three-dimensional reconstruction assessment and surgical plan planning before the operation. Only the intraoperative method was used as the grouping variable. The surgical efficiency, postoperative reactions, and complication rates of the ultrasonic bone knife minimally invasive technique and the traditional bone chiseling and crown splitting method in the extraction of IMTM with the tooth root closely adhering to the IANC were compared. The aim was to clarify the independent clinical benefits of the ultrasonic bone knife technique based on precise preoperative assessment, and to provide more rigorous evidence for the optimization selection of high-risk impacted tooth surgical procedures. The primary hypothesis was that piezosurgery, compared with conventional chisel osteotomy, would significantly reduce operative time, intraoperative blood loss, and early postoperative morbidity (pain, swelling, and trismus) in patients with CBCT-confirmed root–canal intimacy, while also reducing the incidence of inferior alveolar nerve injury.

## 2 Data and methods

### 2.1 Study subjects

We retrospectively collected clinical data from 126 patients with IMTMs whose tooth roots were closely adjacent to the IANC who were treated in the Department of Oral and Maxillofacial Surgery at our hospital between September 2025 and May 2026. All patients underwent CBCT three-dimensional reconstruction before the operation to evaluate the anatomical structure and formulate the surgical plan. The 126 enrolled participants were split into two cohorts of 63 cases each, based on the distinct therapeutic regimens they received: a piezosurgery group and a conventional surgery group. The conventional surgery group underwent traditional bone chiseling and crown splitting for extraction, while the piezosurgery group underwent extraction using the ultrasonic bone knife minimally invasive technique.

Among the participants assigned to the piezosurgery group, there were 34 males and 29 females. Their ages ranged from 19 to 44 years, with a mean age of 28.43 ± 4.90 years. Lesions were present on the left side in 32 patients, whereas the remaining 33 had right-sided lesions. The types of impaction were mesial in 38 cases, horizontal in 20 cases, and inverted in 5 cases. Based on the Pell and Gregory grading criteria proposed in the cited literature (10), the enrolled patients were graded as type B (41 individuals) or type C (22 individuals). The conventional surgery group comprised 32 males and 31 females. The patients' ages ranged from 18 to 45 years, with a mean age of 29.51 ± 4.98 years. In terms of lesion location, 30 patients had left-sided lesions, and the remaining 33 had right-sided lesions. The types of impaction were mesial in 36 cases, horizontal in 22 cases, and inverted in 5 cases. Stratified using the Pell and Gregory grading standard, 43 participants presented with type B manifestations, whereas another 20 subjects fell into the type C category. The baseline demographic and clinical characteristics of the two groups were comparable, with no statistically significant differences between them (all *P* > 0.05; Table 1). The inclusion criteria were as follows: (1) age 18–45 years; (2) preoperative CBCT examination confirmed that the tooth roots were closely adjacent to the IANC, defined as interruption of the cortical bone between the root and the canal or a minimum linear distance of <1 mm at the closest point, consistent with the validated radiographic signs of root–canal proximity described by Rood and Shehab (24) and subsequently confirmed in CBCT studies; (3) no contraindications to extraction surgery; and (4) the patients or their families provided informed consent and signed the surgical informed consent form. The exclusion criteria were as follows: (1) concomitant severe systemic disorders, including cardiac, hepatic, and renal pathologies; (2) coagulation dysfunction; (3) acute inflammation in the surgical area; (4) pregnancy or lactation; (5) inability to cooperate with the surgery due to mental illness. The ethical review board of our medical institution granted authorization to conduct the present trial.

**Table 1:** Baseline characteristics and anatomical classification of the two groups.

Variable	Piezosurgery group (n = 63)	Conventional surgery group (n = 63)	χ^2^/t value	P value
Demographics				
Age (years, mean ± SD)	28.43 ± 4.90	29.51 ± 4.98	1.227	0.222
Sex (male/female)	34/29	32/31	0.127	0.721
Impaction side (left/right)	32/33	30/33	0.127	0.722
Pell & Gregory class (B/C)	41/22	43/20	0.143	0.705
Impaction type			0.149	0.928
Mesial	38	36		
Horizontal	20	22		
Inverted	5	5		
Five-type classification			1.342	0.854
Buccal	15	13		
Lingual	18	16		
Superior	12	14		
Inter-root	11	13		
Intracanal	7	7		
High-risk signs				
Canal compression	23	26	0.301	0.584
Canal deformation	15	19	0.645	0.422

The assignment to the two treatment groups was not randomized but was determined by the treatment period. From September 2025 to January 2026, when the piezosurgery device was unavailable in our department, all eligible patients underwent conventional bone chisel extraction (conventional surgery group). From February 2026 to May 2026, after the institutional procurement of the Woodpecker ULTRASURGERY device and a 2-week team training period, all consecutive eligible patients underwent piezosurgery (piezosurgery group). The surgical team (the same senior surgeons) had at least 10 supervised piezosurgery procedures before performing independent surgeries in the study period, minimizing the effect of the initial learning curve. No selection based on perceived case difficulty occurred because the eligibility criteria remained identical between periods.

### 2.2 Methods

#### 2.2.1 Preoperative imaging assessment

All patients underwent CBCT scanning (RAYSCAN *α*-SM3D) before surgery. The scanning parameters were as follows: tube voltage, 90 kV; tube current, 17 mA; exposure time, 14 s; field of view (FOV), 12 × 8 cm; voxel size, 0.2 mm (isotropic); slice thickness, 0.3 mm; slice spacing, 0.3 mm; and the scanning range covered the body and ascending ramus of the mandible. The 0.2 mm isotropic voxel represents the spatial resolution of the raw acquisition, while the 0.3 mm slice thickness and spacing reflect the reconstruction interval used for clinical review. In our workflow, all quantitative measurements (contact length, width, and height) were performed on 0.3 mm-thick MPR slices, which provided a clinically practical balance between image quality and review efficiency, whereas the 0.2 mm voxel data were used as the source dataset for three-dimensional volume rendering.

The original scan data were imported into the accompanying OnDemand 3D software for three-dimensional reconstruction, generating axial, coronal, and sagittal multiplanar reconstruction (MPR) images, as well as volume reconstruction (VR) stereoscopic images. In our preoperative planning workflow, VR was used solely as a complementary visual aid for stereoscopic spatial orientation, helping the surgical team quickly appreciate the global topographic relationship between the impacted tooth, IANC, and adjacent mandibular ramus. However, because VR does not permit accurate linear measurements or reliable assessment of cortical integrity, all quantitative evaluations—including contact length, contact width, contact height, the five-type anatomical classification, and the identification of high-risk signs (canal compression and deformation)—were performed exclusively on MPR images in the axial, coronal, and sagittal planes. Two senior oral and maxillofacial surgeons jointly reviewed the films and uniformly evaluated the criteria: clearly identifying the location, shape, number of tooth roots, bending direction, three-dimensional spatial relationship (buccal side, lingual side, superior side, inferior side, or surrounding) with IANC, the integrity of IANC cortical bone and morphological changes, and jointly determining the bone removal area, range, depth and tooth body segmentation plan. Both groups of patients underwent the same preoperative assessment process and surgical planning standards to ensure no difference in preoperative diagnostic accuracy between the groups.

Typical evaluation dimensions include measuring the longitudinal contact length between the tooth root and the nerve canal in the sagittal position (Figure 1), measuring the lateral contact width in the axial position (Figure 2), observing the contact point morphology and contact height in the coronal position (Figures 3,4), and classifying the adjacent relationship based on spatial position into five types: buccal, lingual, superior, inter-root, and intracanal. Simultaneously, high-risk signs such as nerve canal compression and deformation were identified (Figure 5). Different classifications correspond to differentiated osteotomy and root division strategies, thereby minimizing intraoperative guesswork and reducing the risk of inadvertent nerve injury by guiding the surgeon's approach based on the specific anatomical relationship. The sagittal, axial, and coronal views of the same representative tooth in three linked orthogonal planes—displayed with identical centering and magnification and cropped to the root–canal region—are shown in Figure 6.

**Figure 1:**
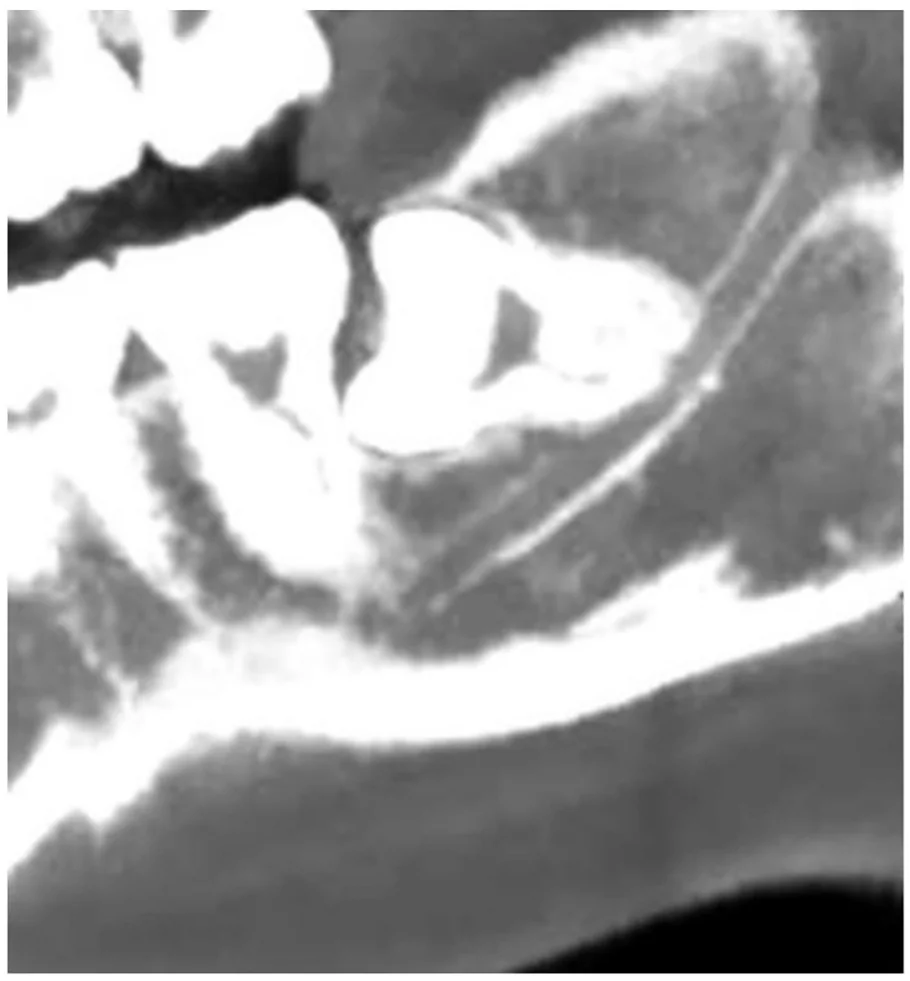
The contact length between the inferior alveolar nerve canal and the root of the mandibular third molar.

**Figure 2:**
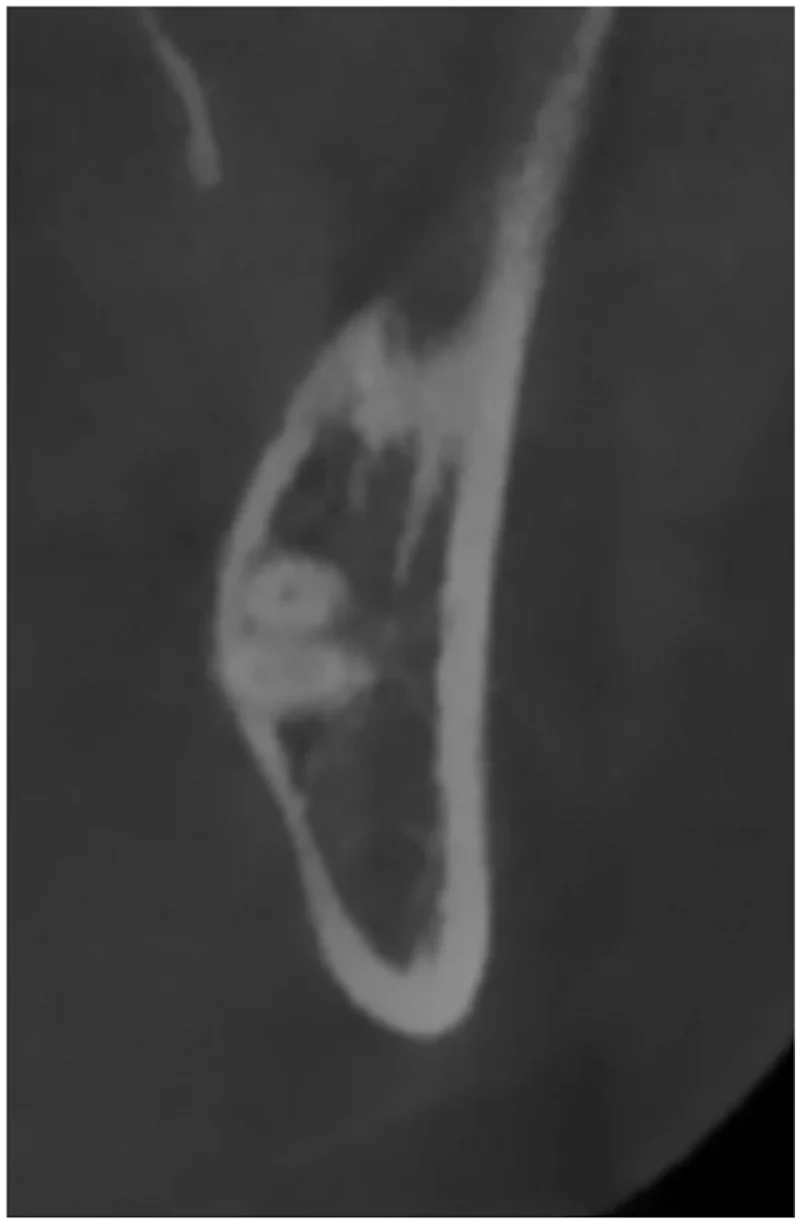
The contact width between the inferior alveolar nerve canal and the root of the mandibular third molar.

**Figure 3:**
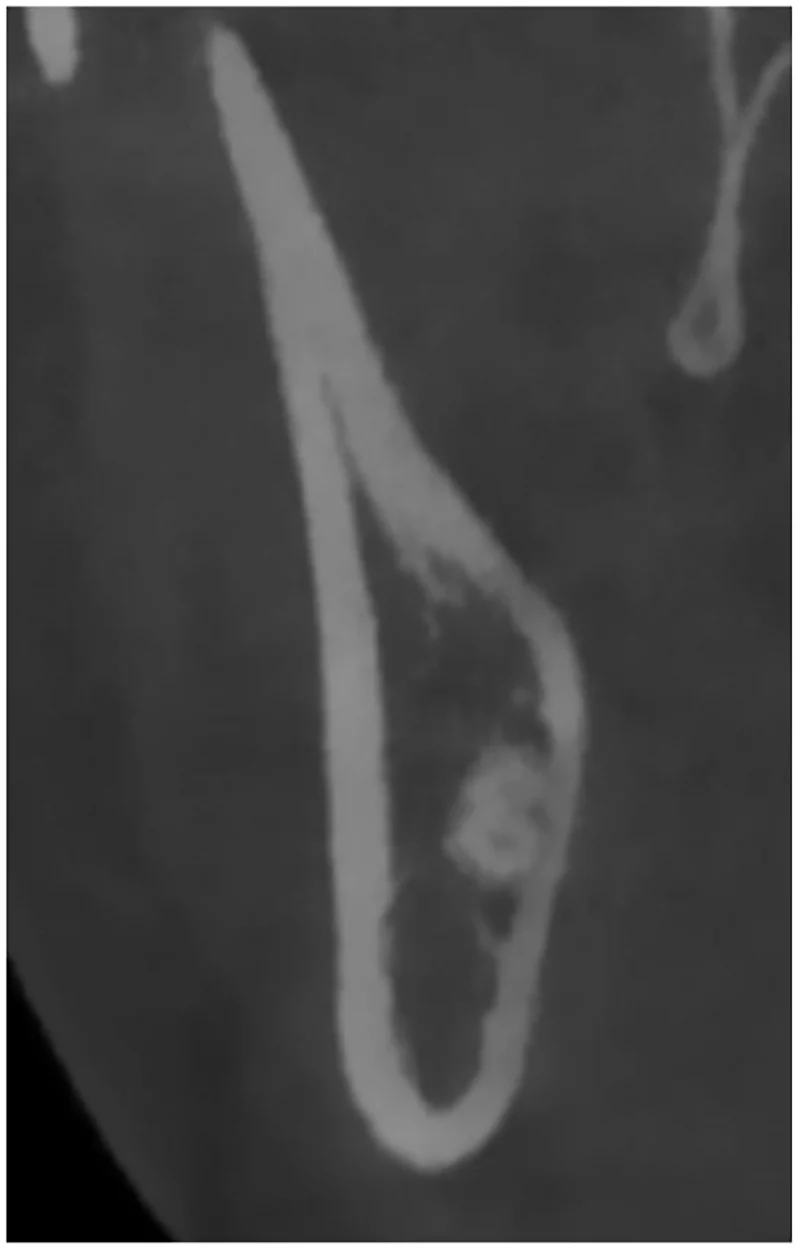
The contact point between the inferior alveolar nerve canal and the root of the mandibular third molar.

**Figure 4:**
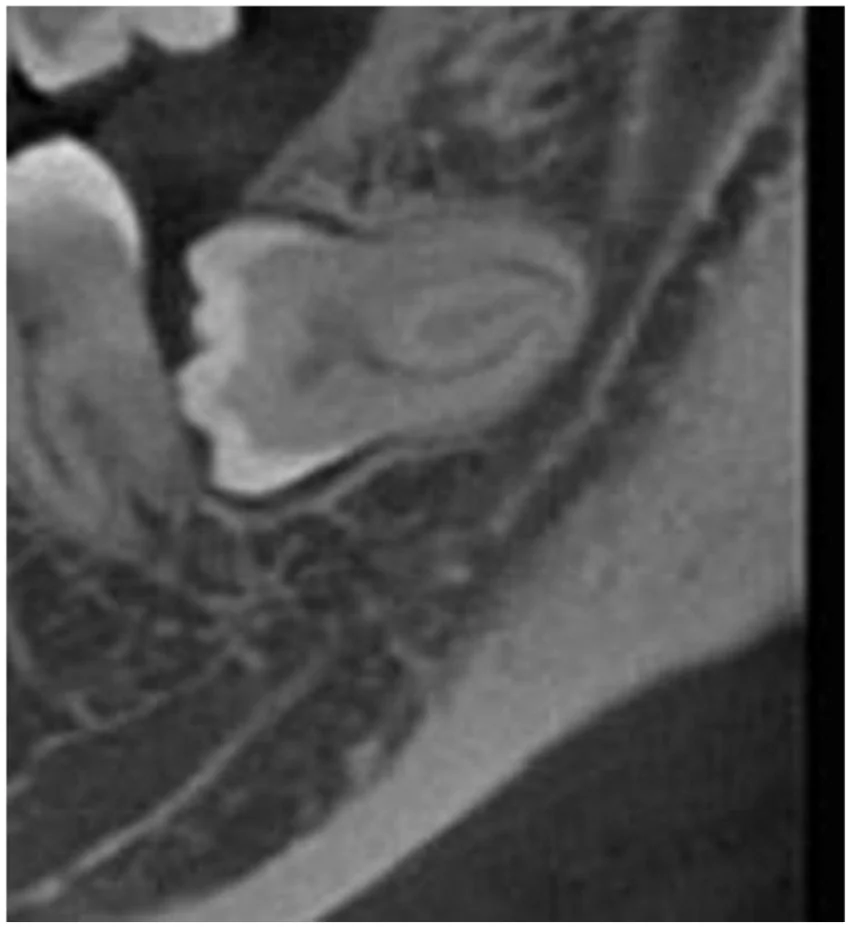
The contact height between the inferior alveolar nerve canal and the root of the mandibular third molar.

**Figure 5:**
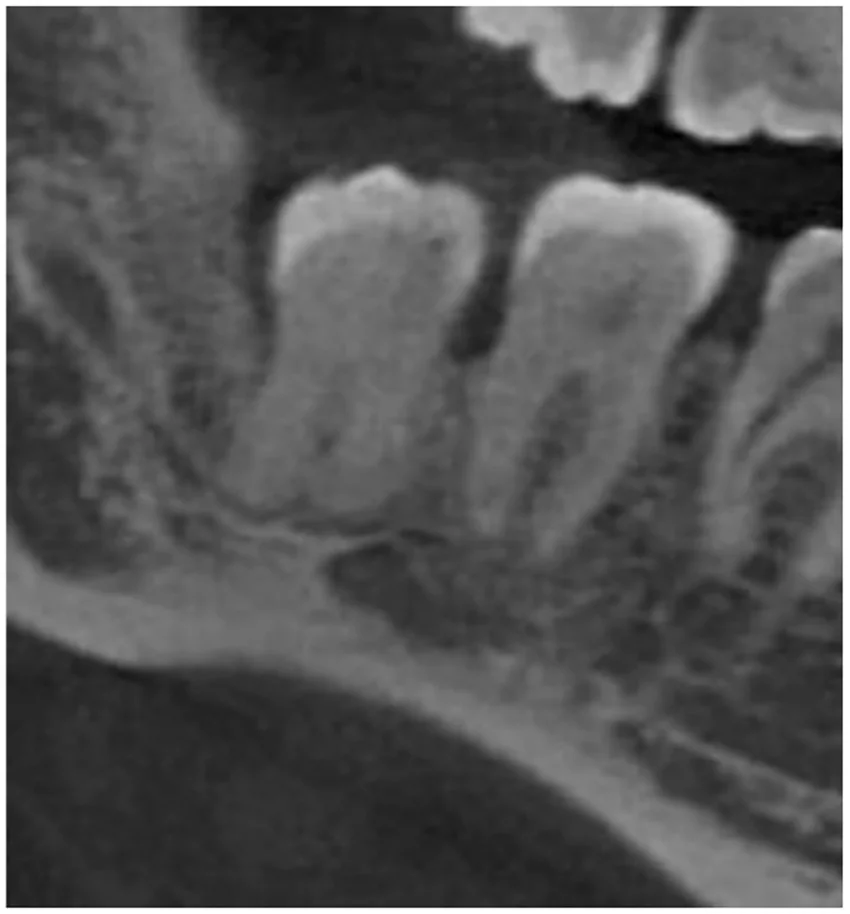
The inferior alveolar nerve canal is deformed due to compression.

**Figure 6:**
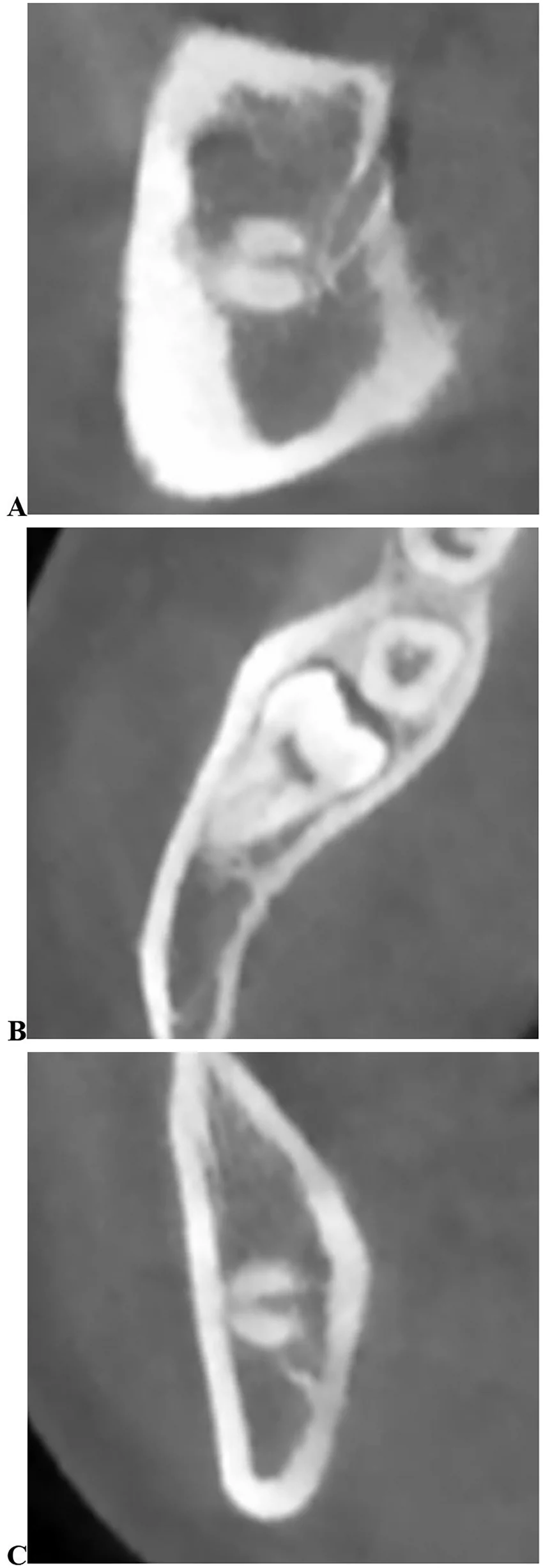
Three linked orthogonal MPR views (sagittal, axial, and coronal) of the same representative tooth, shown with identical centering and magnification and cropped to the root–mandibular canal region of interest; no measurement calipers or graphical overlays are present. **(A)** (sagittal plane): the same representative tooth, showing the longitudinal root-canal contact relationship; the panel is tightly cropped to the region of interest, with no superimposed measurements or extraneous anatomy. **(B)** (axial plane): the same representative tooth, showing the buccolingual position and the contact width between the root and the nerve canal, with centering and magnification matched to panels **(A)** and **(C)**. **(C)** (coronal plane): the same representative tooth, showing the vertical contact height and the root-canal interface morphology, with centering and magnification matched to panels **(A)** and **(B)**.

#### 2.2.2 Surgical method

All operative procedures included in the trial were performed by an identical team of senior physicians with over five years of experience in oral and maxillofacial surgery. In both groups, ipsilateral local anesthesia was achieved via a single inferior alveolar nerve block combined with lingual and buccal nerve infiltration using 2% lidocaine with 1:100,000 epinephrine. The adequacy of anesthesia was confirmed by the absence of pain response to a dental probe before the surgery. The postoperative treatment plan and perioperative nursing pathways were consistent.

The types of surgical incisions and initial flap exposure methods for both groups of patients were identical. After routine disinfection and draping, an angular incision was made, and the mucosa, submucosa and periosteum were cut, and a full-thickness mucoperiosteal flap was elevated to fully expose the buccal and distal bone surfaces of the surgical area. During the operation, care was taken to retract and protect the buccal soft tissue flap with a retractor, avoiding damage to the buccal and lingual nerves. Simultaneously, the left index finger of the hand was used to protect the adjacent teeth and surrounding soft tissues to prevent the instrument from slipping and accidentally injuring them. After completing flap exposure, the two groups differed only in the method of bone removal and tooth crown separation instruments. The details are as follows.

The conventional surgery group underwent traditional bone chiseling and crown splitting. According to the planned bone removal range and depth determined by the preoperative CBCT three-dimensional reconstruction, an appropriately wide bone chisel was selected. Using a single moderate force of hammering, the buccal and distal bones were removed along the predetermined bone removal line. Sufficient osseous tissue must be removed to fully expose the widest coronal dimension of the target tooth and establish a smooth operating channel for the instruments, avoiding excessive bone removal that could damage the lingual bone plate and IANC bone wall. Then, based on the tooth crown resistance direction and developmental groove morphology shown in the preoperative CBCT, the tooth crown was split along the developmental groove using a bone chisel. During the crown splitting process, the placement direction of the bone chisel and the hammering force were strictly controlled to avoid loss of control of the bone splitting direction due to violent operation. After removing the tooth crown in pieces, conventional dental forceps were used, with the mesial alveolar crest as the fulcrum, and the alternating use of lever and wedge forces was employed to gradually loosen the root and completely extract the tooth.

The piezosurgery group underwent the minimally invasive technique using the ultrasonic bone knife. The Woodpecker ULTRASURGERY device was used, and different working tips were selected according to the operative purpose: the US1 working tip was used for bone cortex cutting and precise bone removal, and a dental power system was combined for tooth crown segmentation. The “BONE” mode was selected, and the cooling fluid flow was strictly set at 50 mL/min to ensure that the interface temperature of the bone tissue during the cutting process remained below the safe threshold (<47 ℃), avoiding thermal damage. The bone removal range, depth, and angle determined by preoperative CBCT three-dimensional reconstruction were strictly followed. The tissue-selective cutting characteristic of the ultrasonic bone knife was utilized to precisely remove the buccal and mesial bone tissue using gentle touch techniques. During the cutting process, the working tip was maintained at a 30° to 45° angle with the bone surface to avoid remaining at the same point for more than 5 s. For cases with deeply impacted tooth crowns or significant resistance from adjacent teeth, a dental micro-power system was used to segment the tooth crown, and the pieces were removed in stages to gradually relieve the bone and adjacent tooth resistance. Subsequently, a minimally invasive dental lever was inserted into the periodontal membrane gap pre-expanded by the ultrasonic bone knife, and the rotational wedge method was used to gradually disintegrate the periodontal ligament fibers along the root axis. The tooth root was coronally and buccally displaced and completely extracted. When encountering thin IANC bone walls or bone cortex defects during surgery, the UC1 working tip was used to minimally excise the periapical periodontal ligament tissue, and the contact time with the bone surface was reduced. In necessary cases, a minimally invasive dental lever was used for gentle separation. The patient's facial reactions and any pain complaints were closely monitored to provide real-time warnings of nerve irritation.

After the complete extraction of the two groups of tooth roots, the treatment procedures were identical: the extraction site was carefully inspected, confirming that there were no tooth fragments or residual granulation tissue, the wound was rinsed with normal saline, and the expanded alveolar bone plate was restored. The piezosurgery and conventional surgery groups placed gelatin sponges as needed to support the blood clot according to the wound condition (both groups routinely did not use prophylactic antibiotics). Interstitial suturing of the repositioned gingival flap was performed using a 3-0 silk thread, and the wound was compressed with gauze to stop bleeding. Both groups of patients received unified postoperative health guidance after the operation, including local cold compress within 24 h, warm saline mouth rinsing after 24 h, and oral administration of non-steroidal anti-inflammatory drugs as needed based on the pain condition.

### 2.3 Observation indicators

(1) Surgical-related indicators. The operation time (duration from the incision of the gum to the completion of suturing) was recorded for both groups. Intraoperative blood loss was calculated using the weighing method. blood loss in the piezosurgery group was calculated as (total weight of the postoperative gauze—weight of the preoperative dry gauze)—estimated weight of the gauze that was wetted by the intraoperative known irrigation fluid (subtracting the estimated value of the physiological saline that still wetted the gauze after the suction device was used during the operation), converted to volume at a ratio of 1 g = 1 mL; for the conventional surgery group, the intraoperative blood loss was directly calculated based on the increase in the weight of the postoperative gauze.
(2) Postoperative pain intensity was set as an observation indicator and was evaluated using the visual analogue scale (VAS), as described by Seymour (11). A 100 mm continuous linear scale was used, with “0” (no pain) at the left end and “10” (the worst imaginable pain) at the right end. All patients received standardized instructions regarding the use of the scale before surgery, and the same investigator provided these instructions at each postoperative follow-up. On postoperative days 1, 3, and 7, patients were asked to independently mark their current pain level on the scale, without any suggestive prompts from the attending physicians. A trained assistant blinded to group allocation measured the distance from the “0” end to the patient's mark in millimetres (0–100 mm); this distance was then converted to centimetres by dividing by 10, so that all VAS scores were recorded and are reported on a uniform 0–10 scale (in cm). Each patient completed the scale three times at each time point, and the mean of the three converted values was used for the analysis, so the final score reflects the level marked by the patient.
(3) Degree of facial swelling after surgery. A measurement method using body surface landmark points was adopted. Measurements were conducted 1 day before surgery (baseline) and at 1, 3, and 7 days after surgery at the same time. A medical soft ruler with an accuracy of 0.1 mm was used as the measuring tool. The body position was uniformly set as a sitting position, with the head in the median position and the facial muscles relaxed. The straight-line distance from the lateral point of the same side's corner to the outermost point of the same side's mandibular angle was measured. The difference in facial swelling was used as an evaluation index. The calculation formula was: Facial swelling difference = Measured value at the corresponding time point after surgery—Baseline measured value before surgery. The larger the difference, the more severe was the facial swelling. Each time point was measured three times, and the average value was used for the statistical analysis.
(4) Evaluation of postoperative mouth opening limitation: The inter-incisal distance measurement method was used. Measurements were conducted 1 day before surgery (baseline) and at 1, 3, and 7 days after surgery at the same time. The measuring tool was a vernier caliper with an accuracy of 0.1 mm. The participants were asked to open their jaws as widely as possible under voluntary control. The vertical gap separating the incisal edges of the maxillary and mandibular central incisors was recorded. The difference in mouth opening limitation was used as an evaluation index. The calculation formula was as follows: Mouth opening limitation difference = Baseline measured value before surgery—Measured value at the corresponding time point after surgery. The larger the difference, the more severe the limitation of mouth opening. All measurements collected at each designated time point were repeated in triplicate, and the mean readings were adopted for subsequent statistical calculations.
(5) Incidence of complications: Adverse postoperative events, including inferior alveolar nerve injury, dry socket (alveolar osteitis), surgical site infection, and postoperative hemorrhage, were recorded during the postoperative follow-up.

### 2.4 Statistical methods

All collected clinical datasets were input and processed statistically using SPSS version 22.0 software. Count-based indicators are presented as case numbers plus percentages [n(%)], and intergroup comparisons were performed using the chi-squared test. For the measurement data, the Kolmogorov–Smirnov (K–S) test was conducted to verify whether they followed a normal distribution. Normally distributed measurement data are expressed as $\left(\bar{x}+s\right)$. Intergroup numerical comparisons were conducted using independent samples *t*-tests, whereas paired *t*-test methods were adopted to detect differences in repeated measurements from the same cohort. For the measurement data at multiple time points, repeated measures analysis of variance was used. Before the analysis, Mauchly's test of sphericity was conducted to check whether the spherical hypothesis held true. When the data complied with this sphericity prerequisite, the results of the univariate analysis under the sphericity assumption were used; whenever datasets failed to satisfy the spherical statistical premise, the Greenhouse-Geisser correction was applied to adjust freedom values prior to subsequent statistical examinations. If the interaction effect was statistically significant, pairwise comparisons between groups at different time points and pairwise comparisons between different time points within the group were conducted. Bonferroni correction was applied to adjust the significance threshold for pairwise *post-hoc* comparisons between time points. For the post-hoc pairwise comparisons at individual time points, the significance threshold was adjusted using the Bonferroni correction for three time points (*P* < 0.0167 considered significant). Effect sizes were calculated as Cohen's *d* for between-group comparisons at each time point and as partial *η*^2^ for repeated measures ANOVA factors. Confidence intervals (95% CI) were reported for the main outcome differences. A *P* value <0.05 was considered statistically significant.

## 3 Results

### 3.1 Comparison of surgical-related indicators between the two groups

The operation time of the piezosurgery group was significantly shorter than that of the conventional surgery group, and the intraoperative blood loss was significantly lower (*P* < 0.05), as shown in Table 2.

**Table 2:** Comparison of operative parameters between the two groups.

Group	Cases	Operation time (min)	Intraoperative blood loss (mL)
Piezosurgery group	63	22.76 ± 5.16	17.94 ± 4.20
Conventional surgery group	63	34.33 ± 7.63	33.78 ± 7.18
t value		9.972	15.118
P value		<0.001	<0.001

### 3.2 Comparison of postoperative VAS scores between the two groups

Mauchly's test of sphericity confirmed that the collected dataset violated the assumption of sphericity. Consequently, the Greenhouse-Geisser correction approach was adopted to adjust the corresponding degrees of freedom. Subsequent repeated-measures analysis of variance demonstrated that VAS scores across multiple time points exhibited a statistically significant difference between the two groups (*F*_time_ = 451.509, *F*_between groups_ = 294.939, *F*_interaction_ = 30.945, *P* < 0.05). The VAS scores of both groups showed a continuous downward trend after surgery, and the VAS scores of the piezosurgery group on the 1st, 3rd, and 7th days after surgery were all lower than those of the conventional group (*P* < 0.05), as shown in Table 3 and Figure 7.

**Table 3:** Comparison of postoperative visual analogue scale (VAS) scores between the two groups (cm; 0–10 scale, converted from the 100-mm VAS).

Group	Cases	Postoperative day 1	Postoperative day 3	Postoperative day 7
Piezosurgery group	63	2.98 ± 0.92	2.06 ± 0.64*	0.56 ± 0.15*#
Conventional surgery group	63	5.44 ± 1.51	3.81 ± 0.88*	1.29 ± 0.49*#
Mean difference (95% CI)		2.46 (2.02,2.90)	1.75 (1.48,2.02)	0.73 (0.60,0.86)
Cohen's d		1.96	1.97	1.73
t value		11.024	12.732	8.275
P value		<0.001	<0.001	<0.001

*P < 0.05 compared with postoperative day 1 within the same group; #*P* < 0.05 compared with postoperative day 3 within the same group.

**Figure 7:**
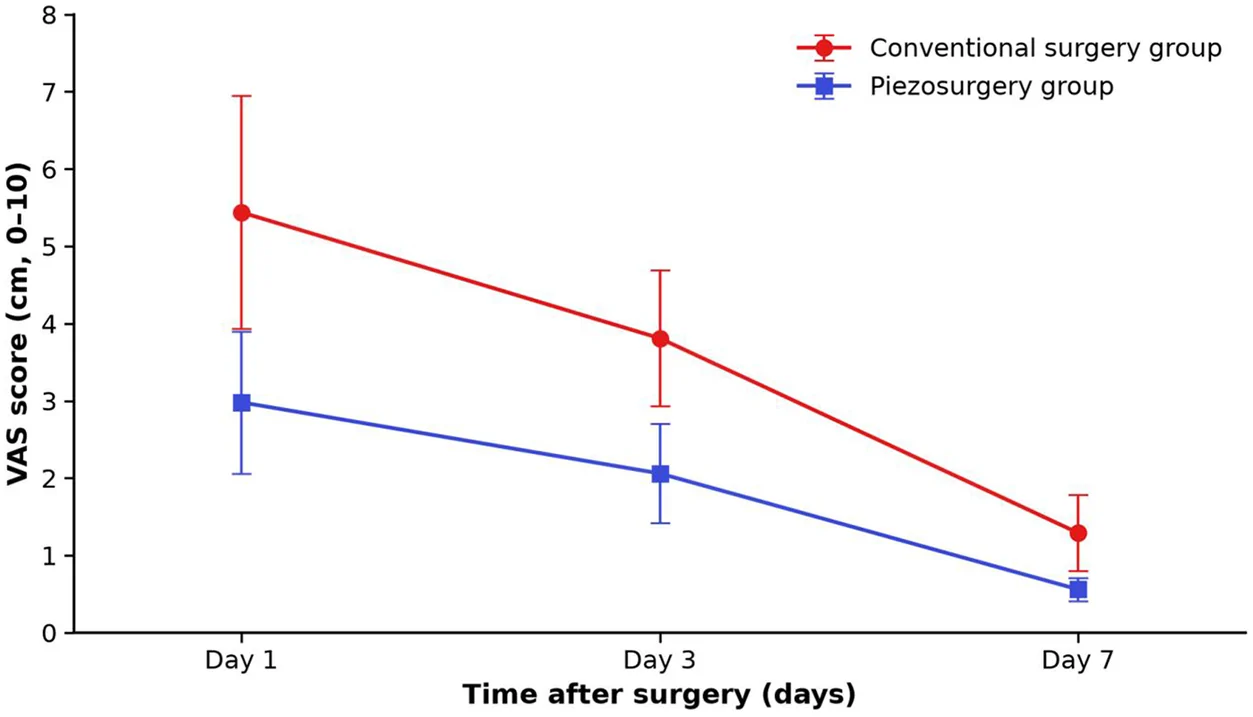
Comparison of postoperative VAS scores between the two groups (cm; 0–10 scale, converted from a 100 mm VAS).

### 3.3 Comparison of the difference in facial swelling after surgery between the two groups

The Mauchly sphericity test results indicated that the data did not meet the sphericity assumption. Therefore, the Greenhouse-Geisser method was used to correct degrees of freedom. The repeated measures variance analysis showed that there was a statistically significant difference in the facial swelling difference between the two groups at different time points (*F*_time_ = 328.621, *F*_between groups_ = 671.732, *F*_interaction_ = 10.973, *P* *<* 0.05). The facial swelling differences in the piezosurgery group at 1, 3, and 7 days after surgery were all significantly lower than those in the conventional surgery group, with mean differences of 3.62 mm (95% *CI*: 3.15–4.09), 3.77 mm (95% *CI*: 3.21–4.33), and 2.32 mm (95% *CI*: 2.01–2.63), respectively, and large effect sizes (Cohen's *d* = 2.71, 2.38, and 2.64), as shown in Table 4 and Figure 8.

**Table 4:** Comparison of postoperative facial swelling differences between the two groups (mm).

Group	Cases	Postoperative day 1	Postoperative day 3	Postoperative day 7
Piezosurgery group	63	4.35 ± 1.10	5.86 ± 1.26*	2.25 ± 0.70*#
Conventional surgery group	63	7.97 ± 1.56	9.63 ± 1.89*	4.57 ± 1.04*#
Mean difference (95% CI)		3.62 (3.15,4.09)	3.77 (3.21,4.33)	2.32 (2.01,2.63)
Cohen's d		2.71	2.38	2.64
t value		15.104	13.233	14.678
P value		<0.001	<0.001	<0.001

*P < 0.05 compared with postoperative day 1 within the same group; #*P* < 0.05 compared with postoperative day 3 within the same group.

**Figure 8:**
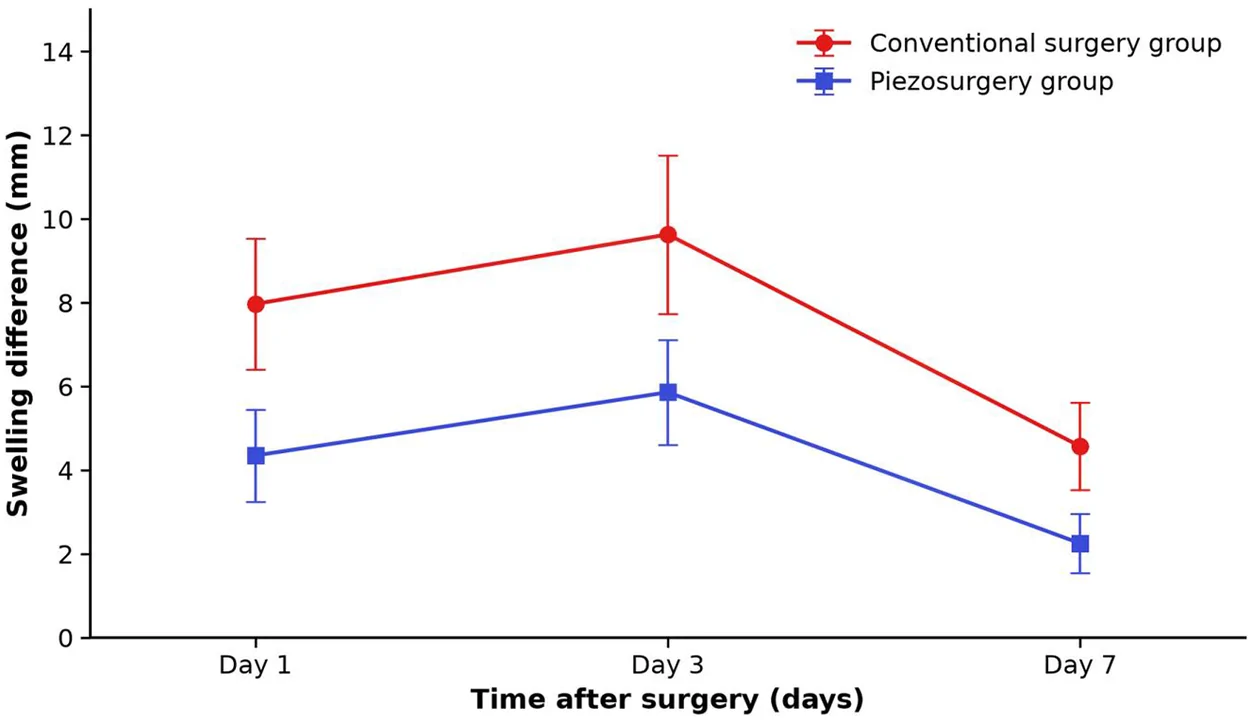
Comparison of postoperative facial swelling differences between the two groups.

### 3.4 Comparison of the difference in postoperative mouth opening restriction between the two groups

The Mauchly sphericity test result indicated that the data did not meet the sphericity assumption. Therefore, the Greenhouse-Geisser method was used to correct degrees of freedom. Repeated measures variance analysis showed a statistically significant difference in the difference of mouth opening restrictions between the two groups at different times (*F*_time_ = 462.382, *F*_between groups_ = 395.906, *F*_interaction_ = 18.287, *P* < 0.05). The trismus differences in the piezosurgery group were significantly lower than those in the conventional surgery group at all three time points, with mean differences of 6.33 mm (95% *CI*: 5.42–7.24), 4.93 mm (95% *CI*: 4.06–5.80), and 3.19 mm (95% *CI*: 2.85–3.53), corresponding to large effect sizes (Cohen's *d* = 2.44, 1.99, and 3.28), as shown in Table 5 and Figure 9.

**Table 5:** Comparison of postoperative trismus differences between the two groups (mm).

Group	Cases	Postoperative day 1	Postoperative day 3	Postoperative day 7
Piezosurgery group	63	8.59 ± 2.19	6.21 ± 1.92*	2.30 ± 0.61*#
Conventional surgery group	63	14.92 ± 2.99	11.14 ± 2.98*	5.49 ± 1.24*#
Mean difference (95% CI)		6.33 (5.42,7.24)	4.93 (4.06,5.80)	3.19 (2.85,3.53)
Cohen's d		2.44	1.99	3.28
t value		13.578	11.061	18.278
P value		<0.001	<0.001	<0.001

*P < 0.05 compared with postoperative day 1 within the same group; #*P* < 0.05 compared with postoperative day 3 within the same group.

**Figure 9:**
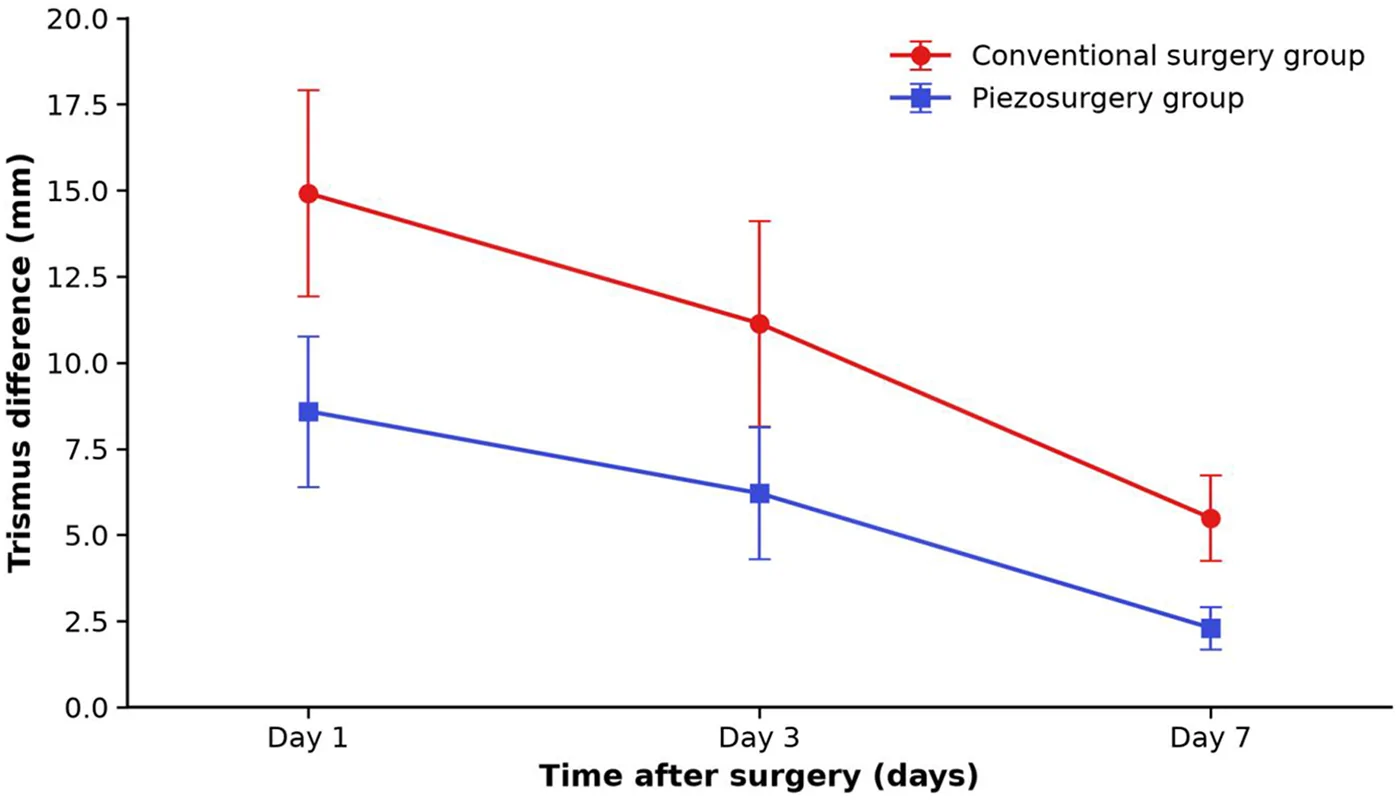
Comparison of postoperative trismus differences between the two groups.

### 3.5 Comparison of the incidence rates of complications between the two groups

The overall complication rate was lower in the piezosurgery group than in the conventional surgery group (4.76% vs. 19.05%, *P* = 0.013) (Table 6). Regarding inferior alveolar nerve injury, one event occurred in the piezosurgery group and six in the conventional surgery group. While this difference is consistent with the neuroprotective effect of piezosurgery, the small number of events (*n* = 7 total) limits the statistical precision of this estimate, and the finding should be interpreted as hypothesis-generating rather than definitive.

**Table 6:** Comparison of complication incidence between the two groups [*n*(%)].

Group	Cases	Inferior alveolar nerve injury	Dry socket	Surgical site infection	Postoperative hemorrhage	Total complications
Piezosurgery group	63	1 (1.59)	1 (1.59)	0 (0.00)	1 (1.59)	3 (4.76)
Conventional surgery group	63	6 (9.52)	3 (4.76)	2 (3.17)	1 (1.59)	12 (19.05)
*χ*^2^ value						6.130
P value						0.013

## 4 Discussion

In cases where the tooth root of the IMTM extraction is closely attached to the IANC, the complex anatomical structure and limited operating space make surgery challenging. The traditional bone chiseling and crown splitting method is widely used in clinical practice because of its simple operation and low treatment cost; however, its operation mode relies on hammering force, which causes significant trauma and limited cutting accuracy. In addition to local reactions such as postoperative pain, swelling, and limited mouth opening, this technique carries a high risk of damaging the inferior alveolar nerve. Unpredictable bone splitting trajectories are to blame for this complication, which may trigger transient or lifelong numbness in the lower lip and greatly impair patients' daily living conditions (12). In recent years, three-dimensional reconstruction using cone-beam CT has gradually become the standard method for the preoperative assessment of complex impacted teeth, reducing surgical blindness at the diagnostic level. The ultrasonic bone knife, a new type of bone-cutting equipment, demonstrates significant technical advantages in minimally invasive and soft tissue protection.

Statistical outcomes from the present trial revealed that the piezosurgery group spent an average of 22.76 ± 5.16 min on surgical procedures, which was less than the 34.33 ± 7.63 recorded for the conventional surgery group. Meanwhile, the volume of hemorrhage generated during surgery was markedly reduced compared with that of the conventional surgery group, suggesting that the minimally invasive technique combining CBCT three-dimensional reconstruction and ultrasonic bone knife can shorten the operation time and reduce the intraoperative blood loss. When the continuity of the root and IANC cortical bone is interrupted, or the root apex is closely adhering to the canal wall, the surgical area is strictly limited within a narrow “safe operation window.” Any bone removal beyond the planned range or uncontrollable bone splitting may directly lead to damage to the nerve sheath, exposure of the nerve bundle, and even rupture. The ultrasonic bone knife achieves micron-level cutting of bone tissue through high-intensity focused ultrasound vibration, without the need for hammering assistance throughout the process, reducing the unpredictable risk of bone fracture line extension in the traditional chiseling method, and can precisely remove the buccal and mesial bone according to the three-dimensional boundaries planned before the operation, avoiding the invasion of the IANC dangerous area due to blind bone removal or uncontrollable crown splitting direction (13). These findings align with the conclusions of Wang et al. (14), whose study showed that the operation time of the ultrasonic bone knife group was shortened by approximately 35% compared to the traditional chiseling group, and the intraoperative blood loss was reduced by approximately 21%. Scolozzi et al. (15) also found that the precise cutting characteristics of the ultrasonic bone cutter are more prominent in low-position impacted wisdom teeth, which can reduce the operational difficulty and improve the surgical efficiency.

From a surgical perspective, CBCT three-dimensional reconstruction provides several actionable insights that directly influence intraoperative safety and efficiency. First, the sagittal view (Figure 1) measures the longitudinal contact length between the root and the IANC, informing the surgeon of the extent of bone removal required along the mesiodistal axis. Second, the axial view (Figure 2) reveals the buccolingual contact width and canal position relative to the root, which determines the direction of instrument insertion and the safe zone for luxation. Third, the coronal view (Figures 3, 4) shows the vertical contact height and morphology of the contact point, which is critical for deciding whether the root can be delivered coronally or requires sectioning. The five-type classification—buccal, lingual, superior, inter-root, and intracanal—enables the surgeon to preselect specific osteotomy and root-division strategies, thereby reducing intraoperative guesswork and minimizing the risk of inadvertent canal entry. In our practice, for example, buccal-type proximity often requires lingual bone plate preservation and buccal corticotomy, whereas inter-root proximity necessitates separating the roots before delivery. These preoperative planning steps, all derived from the same CBCT dataset, are the key links between diagnostic imaging and successful surgical execution. Postoperative pain is the most direct traumatic reaction of patients' subjective feelings and is a core indicator for evaluating the effectiveness of minimally invasive surgical techniques. The results of this study show thatthe VAS scores of the piezosurgery group at 1, 3, and 7 days after surgery were all lower than those of the conventional surgery group, suggesting that the ultrasonic bone knife can alleviate pain level of patients during the postoperative recovery period. From a mechanism perspective, postoperative pain mainly results from bone tissue damage, soft tissue traction and tears, and the release of inflammatory mediators such as prostaglandins and bradykinin after trauma (16). The impact force of the traditional bone chiseling method can be conducted along the bone tissue, causing micro-fractures of the bone trabeculae, periodontal ligament shock, and stress damage in the temporomandibular joint area. Simultaneously, the rough and irregular bone wound surface formed by the splitting of the bone chisel, along with a large amount of necrotic bone debris, will continuously stimulate the intensification of the local inflammatory response. The bone wound surface formed by the ultrasonic bone knife cutting was neat and smooth, with less bone tissue damage, and there was no additional damage to the surrounding soft and hard tissues during the entire process. There is no hammering vibration that causes additional damage to the surrounding soft and hard tissues, and the local inflammatory response after surgery is less, with fewer pain stimulus sources (17). Alotaibi et al. (18) indicated that the incidence of moderate to severe pain 24 h after minimally invasive tooth extraction with an ultrasonic bone knife was lower than that with the traditional method, which is consistent with the results of this study.

Facial swelling and limited mouth opening are the most common local tissue reactions after the extraction of impacted teeth, directly affecting patients' daily functions, such as eating and speaking, as well as their quality of life (19). The results of this study show that the facial swelling of both groups of patients presented a pattern of increasing first and then decreasing after the operation, reaching the peak on the 3rd day after the operation; the differences in facial swelling between the research and conventional groups on the 1st, 3rd, and 7th days after the operation were all smaller; the differences in mouth opening limitation at each time point between the research and conventional groups were also lower, suggesting that the recovery speed of the masticatory muscle function was faster. Facial swelling after surgery is mainly related to an increase in capillary permeability and tissue fluid exudation caused by surgical trauma, whereas limited mouth opening is directly related to the spasm and edema of masticatory muscles, such as the masseter and medial pterygoid muscles, after mechanical stimulation (20). The operation of the ultrasonic bone cutter does not require hammering to transmit force, thereby avoiding shock stimulation of the masticatory muscle group. Moreover, precise bone removal can reduce the range of soft tissue separation and the degree of traction, thereby alleviating tissue edema and muscle spasms. Demirci et al. (21) found that compared with traditional methods, the ultrasonic bone cutter can significantly improve postoperative facial swelling and limited mouth opening. This result is consistent with that of our study, further supporting the clear advantage of the ultrasonic bone cutter in reducing local reactions after surgery.

Statistical analysis of our clinical data demonstrated a lower overall rate of adverse surgical events in the piezosurgery group than in the conventional surgery group. It is worth noting that both groups of patients received the same standard CBCT three-dimensional reconstruction assessment before the operation, and the spatial path of the nerve canal and the condition of the bone wall were clearly identified. Under these circumstances, the nerve injury rate was lower in the piezosurgery group (1/63, 1.59%) than in the conventional surgery group (6/63, 9.52%). Although this difference is clinically meaningful and consistent with the selective tissue-cutting properties of piezosurgery, the small absolute number of events precludes a definitive conclusion. Therefore, we frame this finding as preliminary evidence that warrants confirmation in larger, adequately powered prospective trials. The ultrasonic bone knife has the characteristic of selective tissue cutting, only generating cutting effects on hard bone tissues, and the cutting effect automatically disappears when it comes into contact with soft tissues, such as nerves and blood vessels. Even in high-risk areas with a thin or damaged bone cortex of the nerve canal, it can effectively avoid direct mechanical damage to the nerves (22). Beziat et al. (23)'s multi-center clinical study showed that when the ultrasonic bone knife was applied to the extraction of lower impacted teeth, the temporary injury rate of the inferior alveolar nerve was approximately 1.8%, which was close to the results of this study.

Although CBCT provides indispensable anatomical information for high-risk cases, its routine use for every impacted third molar extraction remains debatable from both cost-effectiveness and radiation exposure perspectives. CBCT delivers a higher effective radiation dose (approximately 26–107 µSv) than panoramic radiography (approximately 4.7–14.9 µSv), and the ALARA (As Low As Reasonably Achievable) principle should guide its application. Therefore, we suggest a stratified approach. CBCT is strongly recommended for cases with clinical or panoramic radiographic signs suggesting root–canal intimacy (e.g., root darkness, loss of the white line, or canal deviation), whereas panoramic radiography may suffice for low-risk cases without such signs. Based on the results of this study, the following stratification recommendations are proposed:

Indications selection. The use of ultrasonic bone scalers is recommended in the following high-risk cases: (1) CBCT confirms direct contact between the tooth root and the IANC (distance = 0 mm) or discontinuity of the cortical bone; (2) Pell and Gregory Class C impaction or horizontally/inversely impacted teeth; and (3) a thin lingual cortical plate or an IANC running between the root bifurcations. Conventional surgical methods may still be considered for medium- and low-risk cases (with a safety distance ≥1 mm). Cost-effectiveness and configuration. The cost of ultrasonic bone scalers and their consumables is relatively high. However, considering the comprehensive benefits such as reduced complications (the absolute risk of nerve injury decreased by approximately 8% in this study), improved surgical efficiency (an average reduction of 11.6 min), and earlier patient rehabilitation, it is recommended that tertiary hospitals with more than 500 cases of impacted teeth surgery per year prioritize the configuration of ultrasonic bone scalers and use them as a routine surgical method for high-risk cases.

Promotion strategy at the grassroots level: Owing to the limitations of equipment price and learning curve, a stratified promotion strategy is recommended: tertiary hospitals undertake the diagnosis and treatment of high-risk cases and technical training; grassroots hospitals can perform the surgery in place for those with medium- and low-risk cases as assessed by CBCT and refer high-risk cases to the superior center. Preoperative CBCT assessment cannot be simplified; surgeons need to accumulate at least 20 cases of medium- and low-risk cases for surgical experience before independently performing high-risk surgeries.

The observation period of this study was set at 7 days after surgery, mainly focusing on the acute local reactions caused by surgical trauma (pain, swelling, and limited mouth opening) and early complications. This study did not track the long-term outcomes of the function of the inferior alveolar nerve (one month and three months or later after surgery) and the quality of alveolar bone tissue healing. This design was mainly based on the following considerations: acute indicators are the most direct reflection of patients' postoperative quality of life and the most sensitive window for demonstrating the advantages of minimally invasive surgical techniques; at the same time, the core value of the ultrasonic bone knife in nerve protection lies in avoiding direct mechanical damage during the operation, while the long-term assessment of nerve function recovery is affected by multiple confounding factors such as individual nerve regeneration ability, age, and underlying diseases, and requires a larger sample size and a longer follow-up period for verification. Beyond the above indicators, only one participant from the piezosurgery group developed transient abnormal sensation related to the inferior alveolar nerve throughout the trial, whereas six such adverse cases were documented in the conventional surgery group. Notable disparities in the overall adverse event proportions were identified across the two patient cohorts. Owing to the relatively small number of nerve injury events, no subgroup analysis of nerve injury type (temporary/permanent) was conducted. Future studies should extend the follow-up period to more than six months, combined with quantitative sensory testing and neuroelectrophysiological examinations, to clarify the differences in the long-term prognosis of nerve injury between the two surgical methods.

In conclusion, in patients with IMTMs closely related to the IANC and evaluated using CBCT, piezosurgery offers significant advantages over conventional bone chisel extraction in terms of shorter operative time, less bleeding, reduced postoperative pain, swelling, and trismus, and a lower complication rate. These benefits are attributable to the precision, selective cutting, and minimal trauma of the ultrasonic osteotome itself, rather than to the CBCT, which was applied uniformly across both groups as a preoperative planning tool. Our findings support the use of piezosurgery as the preferred technique for high-risk impacted third molar extractions when CBCT confirms root–canal intimacy.

This study has several limitations that should be considered when interpreting the results. First, the retrospective cohort design with temporal allocation may have introduced selection bias, including potential confounding by indication and a learning curve effect. Although the baseline characteristics were comparable between the groups and the same surgical team performed all procedures, we cannot entirely exclude the possibility that subtle improvements in surgical skills over the study period contributed to the better outcomes observed in the piezosurgery group. The absence of randomization also precludes definitive causal inferences. Second, this was a single-center study with a limited sample size (*n* = 63 per group), which reduced the statistical power to detect small but clinically meaningful differences, particularly for rare events, such as permanent nerve injury. Third, the follow-up period was limited to 7 days postoperatively, covering only the acute phase of recovery; therefore, long-term bone healing and nerve recovery beyond this period were not evaluated. Fourth, the same investigators who performed the surgeries also participated in the assessment of some postoperative outcomes, which may have introduced a measurement bias. Although VAS scores were patient-reported and swelling and trismus were measured using standardized instruments and clearly defined anatomical landmarks, the outcome assessors were not blinded to the treatment allocation. Future studies should incorporate a blinded outcome assessment, particularly for subjective and instrument-based measurements. Fifth, the imaging assessments and five-type classifications were performed jointly by two senior surgeons rather than independently. Although this approach reflects clinical practice and consensus-based decision-making, it precludes the formal calculation of inter- or intra-examiner reliability (e.g., kappa or ICC). Consequently, the reproducibility of the classification and measurements has not been established and should be considered a limitation of this study. Future studies should incorporate independent assessments by multiple examiners to evaluate and report the reliability statistics. Sixth, the different methods used to estimate blood loss between the groups may have introduced measurement bias, as the correction formula for irrigation fluid retention in the piezosurgery group relied on an estimated rather than a directly measured factor. This limitation should be considered when interpreting blood loss data. Future multicenter randomized controlled trials with larger sample sizes, longer follow-up periods, independent imaging assessments, and blinded outcome assessments are needed to confirm our findings.

## Consent for publication

All have consented to the submission and publication of this manuscript.

## Data Availability

The original contributions presented in the study are included in the article/Supplementary Material, further inquiries can be directed to the corresponding author/s.
